# A new approach to the classification of carcinogenicity

**DOI:** 10.1007/s00204-022-03324-z

**Published:** 2022-06-15

**Authors:** John E. Doe, Alan R. Boobis, Samuel M. Cohen, Vicki L. Dellarco, Penelope A. Fenner-Crisp, Angelo Moretto, Timothy P. Pastoor, Rita S. Schoeny, Jennifer G. Seed, Douglas C. Wolf

**Affiliations:** 1 School of Pharmacy and Biomolecular Sciences, Liverpool John Moores University, Byrom Street, Liverpool, L3 3AF UK; 2 National Heart and Lung Institute, Hammersmith Campus, Imperial College London, London, W12 0NN UK; 3 Department of Pathology and Microbiology, Havlik-Wall Professor of Oncology, University of Nebraska Medical Center, Omaha, NE 68198-3135 USA; 4 Independent Consultant, Silver Spring, MD 20901 USA; 5 Independent Consultant, North Garden, VA 22959 USA; 6 Dipartimento di Scienze Cardio-Toraco-Vascolari e Sanità Pubblica (Department of Cardio-Thoraco-Vascular and Public Health Sciences), Università degli Studi di Padova, Padua, Italy; 7 Pastoor Science Communication, LLC, Greensboro, NC 27455 USA; 8 Rita Schoeny LLC, Washington, DC 20002 USA; 9 Independent Consultant, Alexandria, VA 22301 USA; 10 Syngenta Crop Protection LLC, Greensboro, NC 27419 USA

**Keywords:** Carcinogenicity, Classification, Mew, Approach, Methodology

## Abstract

Concern over substances that may cause cancer has led to various classification schemes to recognize carcinogenic threats and provide a basis to manage those threats. The least useful schemes have a binary choice that declares a substance carcinogenic or not. This overly simplistic approach ignores the complexity of cancer causation by considering neither how the substance causes cancer, nor the potency of that mode of action. Consequently, substances are classified simply as “carcinogenic”, compromising the opportunity to properly manage these kinds of substances. It will likely be very difficult, if not impossible, to incorporate New Approach Methodologies (NAMs) into binary schemes. In this paper we propose a new approach cancer classification scheme that segregates substances by both mode of action and potency into three categories and, as a consequence, provides useful guidance in the regulation and management of substances with carcinogenic potential. Examples are given, including aflatoxin (category A), trichlorethylene (category B), and titanium dioxide (category C), which demonstrate the clear differentiation among these substances that generate appropriate levels of concern and management options.

**Supplementary Information:**

The online version contains supplementary material available at 10.1007/s00204-022-03324-z.

## Concerns over carcinogenicity assessment

Changes in the assessment of the carcinogenic potential of chemicals are being driven by increased levels of understanding of the aetiology and pathogenesis of cancer. In addition, there is a commitment to reduce use of the long-term rodent bioassay (LTRB) and to adopt more relevant and informative tools, such as New Approach Methodologies (NAMs), which can include both in vitro and in vivo studies (Wolf et al. 2019; Cohen et al. 2019; Madia et al. 2019). At the same time there is continued pressure to restrict the use of, or ban, chemicals over potential concerns for specific hazards. For example, the Sustainability Strategy for Chemicals introduced in the EU calls for a “toxic free” environment (EU 2020), although this term is not well defined. The EU apparently uses the concept of Substance of Very High Concern (SVHC) to reach this objective: substances identified as SVHC are subjected to a series of precautionary and mandatory control measures, including ban and substitution with other substances. Classification as a “carcinogen” automatically places a chemical in the SVHC category, which carries the risk of introducing a less well studied replacement that may be of greater concern.

Classification based on the results of the rodent bioassay for cancer and the carcinogenic hazard to humans (United Nations 2019) assumes that carcinogenicity is an intrinsic property of the chemical unrelated to the context of exposure. Although classification guidance suggests that factors such as use, kinetics, species differences, dose, and mode of action (MOA) be considered (ECHA 2017; United Nations 2019), it is unclear how much consideration is given to these additional lines of evidence. Furthermore, their impact on decisions after classification is questionable when downstream risk management decisions are mandated without assessment of risk. While a carcinogenic outcome is claimed to be an intrinsic property of a chemical substance, that is not the case; rather, chemical carcinogenesis is a function of dose, exposure route, and exposure duration and the response may differ between species (McCarty et al. 2020). Thus, carcinogenicity is not an intrinsic property of the molecule but rather a function of the chemistry, the exposure scenario, and the species being exposed.

The stated purpose for classification is to identify intrinsic properties of a chemical substance and then to inform the public as to potential hazards, so that informed decisions can be made on use and risk mitigation. However, concerns have been expressed that the EU Sustainability Strategy provides an overly simplistic answer to a complex question (Boobis et al. 2016; Doe et al. 2019). It is appealing to attempt to distinguish between Substances of Very High Concern and substances of lesser concern based on identifying a specific hazard, considering it as an intrinsic property of the chemical such as carcinogenicity, without characterising the hazard, its MOA, dose–response, and exposure, and hence, the risk potential. In this way, decisions are made without weighing all the evidence or considering the consequences. We have previously suggested that the controversy around hazard-based and risk-based classification schemes lies in the way that hazard is codified (Doe et al. 2021). We proposed that there are three levels of hazard codification:

- Level 1: Hazard identification based on the presence or absence of a class of adverse effect; Yes or No binary choice (limited banding); No quantification,
- Level 2: Compartmentalization of hazard first by nature of the adverse effect and then by potency of the agent by banding with several categories (typically 3-5); semiquantitative in nature.
- Level 3: Description of the nature of an adverse effect and the derivation of a health-based guidance value (e.g. acceptable daily intake, reference dose, derived no effect level) to establish acceptable levels of exposure. This entails no banding but continuous dose response determination for risk assessment, and it is fully quantitative.

Currently, Levels 1 and 2 are used in classification and Level 3 in risk assessment. The expressed concerns about the EU Sustainability Strategy (Herzler et al. 2021) and about schemes such as IARC classification (Boobis et al. 2016) are, amongst others, linked to the severe limitations of Level 1 (binary) codification. The appeal of these classification schemes is in apparent simplicity and the supposed clarity that they offer in providing guidance to users and consumers over a range of situations. This is the so called “generic risk assessment” idea, that is focused neither on dose response nor a specific exposure scenario. While Level 2 (Hazard banding) codification reflects differences in chemical severity of effect and potency, Level 1 (binary) codification is overly simplistic and misleading. It fails to consider differences in potencies that range across 7 orders of magnitude for substances classified as “carcinogens” (Gold et al. 1989). This wide range in potency is driven by the diversity of chemically-induced biological effects that may result in an increased incidence of cancer, and the associated individual potency of the chemicals that could induce these effects.

## Current models of carcinogenesis

The adverse outcome pathway (AOP) concept has been used to develop a unifying model for carcinogenesis within which the range of biological effects can be placed. An example is the Dynamic Cancer Model (Harrison and Doe 2021) that describes carcinogenesis as an ongoing process leading to cancer formation in 40% of humans during a typical lifetime (Sasieni et al. 2011). The process starts with mutations in a stem cell undergoing cell division, an event that is very frequent. It has been estimated that 2 × 10^8^ cells with mutations that could lead to cancer are produced in a human lifetime (Harrison and Doe 2021). Most of these mutated cells do not survive or complete the process of tumorigenesis. The process is governed by three rates:

- Number of stem cell divisions per period of time
- Rate of mutation per cell division to produce a cancer- associated mutation
- Rate of cancer-capable cells surviving and progressing to finally become a cancer

Chemicals may affect the carcinogenesis process and lead to changes in tumor incidence by modifying one or more of these rates.

## Bringing NAMs into carcinogenicity assessment

The current binary classification schemes for carcinogenicity are based on the strength of evidence that an increased incidence of neoplasms has occurred in either epidemiology or long-term rodent bioassays. There is some grading based on the strength of the evidence, but there is no consideration of chemical dose–response or relative potency. There is a concerted effort to develop new lines of evidence for assessing carcinogenicity potential, which is motivated by a desire to strengthen the evidence from epidemiology and LTRB. In addition, it is acknowledged that epidemiology and LTRB take a long time, are expensive and, for LTRB, involve many laboratory animals and is often not reliably predictive of human carcinogenicity (Gaylor. 2005; Cohen et al. 2019). Evidence on the potential for chemicals to modify cancer risk can be drawn from a wide range of sources, including in silico-based structure activity, in vitro studies and short-term appropriately designed in vivo studies (Madia et al. 2019). The ongoing challenge is to find ways to interpret and integrate these lines of evidence into a relevant overall assessment (Cohen et al. 2019; Luitjen et al. 2020; Madia et al. 2021).

Inspired by the idea of the Hallmarks of Cancer (Hanahan and Weinberg 2000, 2011), the Key Characteristics of Cancer (KCC) (Smith et al. 2016; Guyton et al. 2018) concept has been developed as an attempt to identify and organize new lines of evidence for assessing carcinogenicity. Smith et al. (2016) analysed the biological effects of chemicals classified as known human carcinogens and deduced that they show one or more of 10 key characteristics (KCCs). Tice et al. (2021) reviewed the KCCs with the intent of developing an integrated approach to testing and assessment (IATA) of carcinogenic potential using NAMs. Their conclusion was that the KCCs lack specificity for carcinogenicity as they are also involved in disease processes that are not related to cancer. We note that the KCCs are not key (as defined by WHO for MOA (Boobis et al. 2006) and by OECD for AOP (OECD 2016)) because each one is not essential solely for carcinogenesis. Nor are they characteristic because they do not selectively describe or relate only to modes of action that result in carcinogenesis. There is also no guidance on how the KCCs could be used in a quantitative manner.

Madia et al. (2021) have tried to map a range of NAMs against the KCCs to construct an IATA for carcinogenicity assessment. A similar approach is being used by the OECD for an IATA for non-genotoxic carcinogenicity (Jacobs et al. 2020). These attempts have highlighted the fact that several of the KCCs lack specificity. It is difficult, if not impossible, to conclude whether or not a chemical with one or more KCC is a “carcinogen” or even a “potential carcinogen”. Strict use of the term “Key Characteristic” would demand that a chemical show all 10 KCCs, thus severely limiting the number of chemicals identified as “carcinogens.” Loose application of the term would allow any chemical showing at least 1 KCC to be identified as a “carcinogen”. This would extend the “carcinogen” category too widely to be practicable or scientifically defensible. No scheme has yet been proposed and published in which the requisite number of KCCs is more than one but less than 10, nor on the criteria that would be used to determine which and how many KCCs should be used to make that determination. KCCs, used judiciously, do have a role to play as lines of evidence in assessing carcinogenic potential, but only when combined with other relevant information.

## What are we trying to do?

So how can the complexity of the carcinogenesis process be reconciled with the desire for a simple but still predictive and useful classification scheme? The first step is problem formulation, the posing of the appropriate question. Firstly, it is necessary to identify those chemicals that are potentially carcinogenic. But identifying any possibility of carcinogenic potential without further qualification is of limited benefit when giving guidance on the use of chemicals. Therefore, the question could be framed as: “Can we develop a process for assessing carcinogenic potential.

I. That is based on existing knowledge, which can use established and/or new methodologies, and
II. That will provide useful guidance to product developers, users, and consumers on the application of chemicals so that excess cancer will not occur?”

It seems that what is required to answer affirmatively to the first part of this question is already in place. Current knowledge has enabled a framework to be devised based on the AOP concept supporting the integration of existing and new lines of evidence (Cohen et al. 2019; Harrison and Doe 2021; Jacobs et al. 2020: Madia et al. 2021). However, tools to answer the second part of the question are lacking. This requires consideration of what useful guidance would look like, which would, in turn, translate into appropriate risk management measures. The two extremes dictated by a Level 1 (binary) codification scheme of a chemical being either “carcinogen” or “not a carcinogen” are not useful. Could a more reliable and useful Level 2 (hazard category bands) codification scheme be developed which could take better advantage of the new lines of evidence provided by NAMs including potency considerations?

## A new approach carcinogenicity classification scheme

Are all identified carcinogenic hazards the same? Clearly not. We have noted the seven orders of magnitude of differences in potency that have been observed in LTRBs. There are also differences in the MOA(s)/AOP(s), which impact time to onset, reversibility of pre-neoplastic changes, and other properties that could be used to better characterize the hazard and, thus, suggest more appropriate risk management measures (Harrison and Doe 2021). These include whether there is an increase in the number and nature of background neoplasms or new neoplasms, and whether the increase in neoplasms is preceded by or is a consequence of toxicity or other biological effects. This points to a need for more sophisticated characterization of potential human carcinogenic hazard.

A simple yet informative Level 2 hazard codification scheme for carcinogenicity would have at least three categories. These categories should provide useful information and clarity on how to use the lines of evidence to assign a substance to the correct category. There should also be a relationship between what the lines of evidence indicate and that any decisions are based on scientific knowledge based on the interpretation from the categorization.

The Dynamic Cancer Model (Harrison and Doe 2021) provides a route to devising such a scheme. The first part of the process is to consider the evidence that the chemical is capable of modifying stages of the carcinogenesis pathway, and, if so, at what stage(s) and by what process(es). The stages and processes that are affected can be grouped into three broad categories based on MOA (see Table 1).

**Table 1:** Categories of mode of action

	Mode of action	Toxicity as a key event
Primary (Direct)	Induces mutations leading to neoplasm formation	No toxicity necessary
Secondary (Indirect)	Modifies other stages of the carcinogenicity pathway	There may be accompanying toxicity
Tertiary (Collateral)	Causes toxicity which leads to modifications of stages of the carcinogenicity pathway	Toxicity required

Evidence on the MOA can be gained from in vitro or short-term in vivo studies that measure effects which can modify the incidence of cancer as described in the scheme proposed by Cohen et al (2019).

Primary or direct-action: the chemical exposure results in mutations that can lead the cell to start the process of developing into a neoplasm or enhance mutations in genes acting at later stages of the carcinogenic process. There is not necessarily a need for accompanying toxicity or other effects based on chemical exposure, but they may occur. Primary or direct action would be assessed by a battery of genotoxicity studies.

Secondary or indirect-action: the chemical exposure leads to a change in the incidence of cancer by modifying other (non-direct mutation) parts of the pathway in a specific way. This essentially increases the rate of carcinogenesis that is already occurring. This includes the direct stimulation of cell division (mitogenesis) leading to an increase in stem cell division thereby increasing the probability of mutated cells, such as receptor activation. It also includes inhibition of repair mechanisms or modification of the tumor microenvironment. Toxicity is not a key event. Secondary or indirect action would be assessed by in vitro or in vivo studies that could detect effects such as immunosuppression, endocrine effects leading to cell proliferation, cell proliferation studies, or histopathology indicating these changes. There would also be evidence of lack of genotoxicity.

Tertiary or collateral action: exposure to the chemical causes toxicity that leads to a change in other parts of the pathway, resulting in an increase in the rate of carcinogenesis. This may be caused by selective toxicity, for example, causing the death of cells leading to consequent regenerative repair with an increase in stem cell divisions. Tertiary or collateral action would be assessed by in vitro or in vivo studies that are indicative of toxicity leading to cellular damage and replacement. There would also be evidence of lack of genotoxicity.

Care should be taken not to use evidence from excessively high doses either in vitro or in vivo, which have little relation to human exposures or to internal concentrations derived from relevant exposures, as they may produce anomalous results (Bogert et al. 2021). Examples of the evidence that can be used to assess MOA are explored in section 6 of this paper, “Examples of using the new approach carcinogenicity classification scheme”.

Potency is also a necessary consideration as part of the categorization process. Evidence from in vitro data must be extrapolated to an in vivo concentration and related effects (IVIVE) so that predictive models can be developed. These models will be useful in establishing the relationship between the in vitro concentration and the exposure conditions that would result in a relevant in vivo concentration producing a biologic response. Evidence from appropriately designed and conducted short term in vivo studies (e.g. cell proliferation) could help in characterizing the dose response curve (Cohen et al. 2019). In keeping with developing a simple Level 2 hazard codification scheme, the potency could be designated as high, medium, or low. The boundaries between the categories need to be set with care, but a starting point for consideration could be the EU Specific Concentration Limits (EC 2019), which are derived boundaries for carcinogenicity via the oral route:

- High potency: point of departure < 1 mg/kg bwt/day
- Medium potency:1 mg/kg bwt/day < point of departure < 100 mg/kg bwt/day
- Low potency: point of departure > 100 mg/kg bwt/day.

Potency for other routes of exposure and physical form can be derived using the equivalency factors in the EU CLP guidance (ECHA 2017) as shown in Table 2.

**Table 2:** Potency category boundaries for oral, dermal and inhalation routes

	Oral mg/kg bwt	Dermal mg/kg bwt	Gas ppm (v/v)	Vapour mg/l (m/v)	Particulate mg/l (m/v)
High	< 1	< 2	< 5	< 0.2	< 0.02
Medium	1–100	2–200	5–500	0.2–20	0.02–2
Low	> 100	> 200	> 500	> 20	> 2

The categories of MOA and of potency could then be combined as shown in Table 3 to give an overall classification for carcinogenicity.

**Table 3:** Overall classification incorporating mode of action and potency

	Primary—Direct	Secondary—Indirect	Tertiary—Collateral
High Potency	A	A	B**
Medium Potency	A	B	C
Low Potency	B*	C	C

*Strong evidence would be required to designate a Primary-direct to be low potency and thus in Cat B

**Would be classified in the EU as Category 1 for repeat dose toxicity

Each classification would have different implications for the use of chemicals and their risk management. In broad terms, Category A substances would require stringent risk management that may also include the current EU “cut-off” approach used for plant protection products or be based on worst case assumptions of dose response (e.g., linear extrapolation, MOE > 10,000). Category B substances would require health-based guidance values based on the effect(s) that lead(s) to the modification of carcinogenesis to inform appropriate choices for risk management. Guidelines for inclusion in products containing more than one chemical or for use in particular sectors could be devised based on consideration of the chemical potency. Category C substances also require risk management informed by health-based guidance values based on studies, including NAMs that assess repeat dose toxicity, for instance the guidelines used for specific target organ toxicity after repeat dosing (STOT RE).

## Examples of using the new approach carcinogenicity classification scheme

The proposed scheme can use traditional sources of in vivo data and can accommodate information from NAMs. We evaluated this approach using it to classify several chemicals in a series of case examples. Information about each chemical was obtained from credible publicly available sources that included summary information on the results of epidemiology and/or LTRBs and information on MOA, including genotoxicity. There was no reinterpretation of conclusions presented in the information sources, and these examples are for illustrative purposes only and should not be viewed as definitive evaluations.

The assignment to the MOA category proved to be fairly simple. Assignment to the Primary-Direct category used the results of genotoxicity assessments, with in vivo positives given most weight. Chemicals that showed no genotoxicity were assigned to the Secondary-Indirect category unless there was evidence that target organ toxicity could reasonably be associated with the neoplasms. Potency was determined by determining a point of departure for an increase in cancer incidence or for an effect from MOA assessment that is correlated or predictive of an increase in cancer.

The detailed evaluations are available in Supplementary Data to this paper, and brief summaries are included in this section.

**Aflatoxin B-1** (NIEHS 2021; Cullen et al. 1987): shown to be associated with an increase in cancer in a wide range of species, including humans. There is evidence of genotoxicity in vitro and in vivo. Aflatoxin B-1’s MOA is, therefore, a primary-direct. Points of departure can be obtained from the animal studies; one such value is 50 µg/kg, resulting in a category of high potency. Primary-direct MOA and high potency would place aflatoxin B-1 in Category A and it would be subjected to restrictive risk management actions.

**Benz[*a*]anthracene** (IARC 2022a): shown to be associated with tumours in mice. Evidence of in vitro and in vivo genotoxicity. Benz[*a*]anthracene’s MOA is therefore primary-direct. Points of departure can be obtained from animal studies; a value of 0.0002% solution applied to skin equivalent to 10 µg/kg resulting in a category of high potency. Primary-direct MOA and high potency would place Benz[*a*]anthracene in Category A and subjected to restrictive risk management actions.

**B-RAF inhibitors** (Wisler et al. 2011): a category of pharmaceuticals used in the treatment of melanoma. B-Raf is the main activator of the mitogen-activated-protein kinase (MAPK) pathway. B-Raf inhibitors induce early transcriptional changes consistent with activation of the PI3K/AKT and ERK/MAPK pathways driving unchecked cell proliferation, resulting in marked tissue hyperplasia that can progress to carcinoma within a short time frame. They are not genotoxic in a range of in vitro and in vivo assays resulting in their MOA being secondary-indirect. The B-Raf inhibitors lead to tumours in only 28 days in rats dosed at 30 mg/kg indicating their potency is high because of the short duration of dosing required. Secondary-indirect MOA coupled with high potency places them in category A and subjected to restrictive risk management actions.

**Dichloroethane** (ECHA 2022a, b): associated with tumours in oral and inhalation long term bioassays. There is evidence that dichloroethane is not genotoxic in in vitro and in vivo assays. There were no treatment-related changes in organs where there were tumours. As a default, it would be prudent to place dichloroethane into the secondary-indirect category. A point of departure can be derived from the long term bioassay study of a LOAEL of 57 mg/kg placing dichloroethane into the mid-potency category. Secondary-indirect MOA and medium-potency would place dichloroethane into Category B where a health-based guidance value derived from the long term bioassay should be used for risk assessment.

**Hydroquinone** (McGregor 2007): Hydroquinone has been shown reproducibly to induce benign neoplasms in the kidneys of male F344 rats dosed orally either by gavage or diet. All renal tubule adenomas and all cases of renal tubule atypical hyperplasia occurred in areas of severe or end-stage chronic progressive nephropathy. Hydroquinone is considered to be non-genotoxic. Non-genotoxicty and the presence of toxicity in the organs where tumours were seen indicate tertiary-collateral MOA. A point of departure of LOAEL 25 mg/kg in the long term bioassays would place hydroquinone into the medium potency category. It has been concluded that the MOA is not relevant to humans, but if the MOA were to be considered to be relevant the tertiary-collateral MOA and medium potency would place Hydroquinone into Category C where a health based guidance value should be set on the underlying toxicity, in this case renal toxicity.

**Linuron** (EFSA 2016): Increased incidence of Leydig cell tumours, uterine adenocarcinoma and ovarian (granulosa/thecal cell) tumours were present at higher dose levels in rats whereas an increase in hepatocellular adenoma was observed in mice. Linuron has antiandrogenic properties, which is consistent with the major effects being in testis, uterus and ovary. There is evidence that linuron is not genotoxic. Tumour induction is probably caused by increased cell division as a compensatory mechanism for antiandrogen effects, indicating tertiary-collateral MOA. A point of departure is 6.5 mg/kg NOAEL in mice indicating medium potency. The tertiary-collateral MOA and the medium potency would place linuron in Category C, where a health-based guidance value should be used based on the underlying toxicity, in this case anti-androgenicity.

**Ochratoxin** A (Pfohl-Leszkowicz and Manderville 2007): is associated with kidney tumours in rats and there is some evidence in humans. There is frank kidney toxicity in rats and indications of kidney toxicity in humans. The genotoxicity of ochratoxin A is controversial. For the purposes of exploring how the new approach carcinogenicity scheme would handle a compound with a tertiary-collateral MOA with high potency, ochratoxin has been evaluated as having evidence of non-genotoxicity. There is evidence of both carcinogenic activity and toxicity at 70 µg/kg resulting in high potency. The tertiary-collateral MOA, assumed for this exercise, and high potency would place ochratoxin A in Category B, where a health-based guidance value would be set based on the effect of concern, in this case renal toxicity. With activity at 70 µg/kg, a health-based guidance value would be correspondingly very low and result in strict risk management actions. The substance would also be in the highest category of concern for repeat dose toxicity.

**Titanium dioxide** (inhalation) (Kuempel and Ruder 2018): Inhalation studies in rats reported increases in lung tumours. Most evidence suggests that TiO_2_ and other poorly soluble low toxicity (PSLT) particles elicited lung tumors develop via a mechanism involving chronic inflammation, cell proliferation, and oxidative stress. Overloading of lung clearance is accompanied by pulmonary inflammation, production of reactive oxygen and nitrogen species, depletion of antioxidants and/or impairment of other defense mechanisms, cell injury, cell proliferation, fibrosis, and eventually cancer. The MOA would be categorized as tertiary-collateral. A point of departure can be derived from a NOAEL of 0.05 mg/l resulting in medium potency. Tertiary-collateral MOA and medium potency would place titanium dioxide by inhalation in Category C, where a health-based guidance value should be used based on the underlying toxicity, in this case pulmonary inflammation.

**Trichloroethylene** (ECHA 2014): the experimental animal results indicating evidence of carcinogenicity of trichloroethylene in humans were principally the significant increases in kidney tumours in rats, pulmonary tumours in mice and testicular tumours in rats. No consistent histological changes have been seen in these organs to indicate a tertiary-collateral MOA. There is conflicting evidence of genotoxicity, and it is not possible to exclude primary-direct MOA. A point of departure can be derived from the NOAEL of 500 mg/kg NOAEL in a long term oral bioassay, which indicates low potency. Primary—direct MOA and low potency would place trichloroethylene in Category B, which would require health-based guidance value based on the effect underlying the carcinogenicity, in this case low potency genotoxicity.

The results of the categorization are shown in Table 4.

**Table 4:** Categorization of chemicals using the New Approach Classification Scheme Evaluations based on oral studies except where indicated

	Primary—Direct	Secondary—Indirect	Tertiary—Collateral
High Potency	A Aflatoxin B-1 Benz[*a*]anthracene (dermal)	A B-RAF Inhibitors	B Ochratoxin A*
Medium Potency	A	B Dichloroethane	C Linuron Titanium Dioxide (inhalation) Hydroquinone
Low Potency	B Trichloroethylene	C	C

*The genotoxicity of ochratoxin is controversial. For the purposes of exploring how the new approach carcinogenicity scheme would handle a compound with a tertiary-collateral MOA with high potency, ochratoxin has been evaluated as having evidence of non-genotoxicity

**Table 5:** Comparison of outcomes from EU classification using the GHS scheme (GHS/EU Cat), classification by IARC (IARC Cat) and the New Approach Classification Scheme (NAC Cat)

Chemical	GHS/EU Cat	IARC Cat	NAC Cat	MOA Cat	Potency Cat
Aflatoxin B1	1B	1	A	P	H
Benz[*a*]anthracene	1B	2B	A	P	H
B-RAF inhibitors			A	S	H
Trichloroethylene	1B	1	B	P	L
Dichloroethane	1B	2B	B	S	M
Ochratoxin A		2B	B	T	H
Linuron	2		C	T	M
Hydroquinone	2	3	C	T	M
Titanium Dioxide (Inhalation)	2	2B	C	T	M

The components of the NAC Cat are shown for potency (Potency Cat High, Medium, Low) and for Mode of Action (Primary, Secondary, Tertiary)

It is interesting to compare (see Table 5) the outcomes from the use of our New Approach Classification scheme to the results through classification using the GHS scheme as used in the EU (ECHA 2022a, b) and to the classification that the IARC monograph program uses (IARC 2022b). The EU/GHS scheme provides no distinction between aflatoxin B1 and benz[*a*]anthracene (high potency-direct) and trichloroethylene (low potency primary-direct) and 1, 2-dichloroethane (medium potency secondary-indirect), placing them all in Category 1B. Our new approach scheme placed the high potency primary-direct agents in Category A and the low potency primary-direct agent (trichloroethylene), the medium potency secondary-indirect agent (dichloroethane) and the high potency tertiary-collateral agent (ochratoxin A) in Category B. Linuron, hydroquinone and titanium dioxide (medium-potency tertiary-collateral agents) were placed in Category 2 by EU/GHS and in Category C by the new approach classification scheme. The IARC scheme placed aflatoxin and trichloroethylene in Category 1 and benz[*a*]anthracene, dichloroethane, ochratoxin B and titanium dioxide in Category 2B, reflecting the strength of the evidence rather than the level of concern based on potency and exposure levels. It is difficult to reconcile the potent genotoxicant benz[*a*]anthracene and a basically inert substance like titanium dioxide having the same IARC classification. It is also difficult to reconcile the two potent genotoxicants, benz[*a*]anthracene and aflatoxin, being classified differently by IARC. The scheme we propose gives a much clearer indication of their relative hazard potential and will better inform risk management decisions.

## Conclusions

Simplistic approaches to complex questions are usually incorrect—carcinogenicity is too complex to be the subject of a Level 1 binary hazard codification scheme. A Level 1 binary scheme does not take into account the range of MOAs and potencies we have referred to in this paper. Thus, a level 1 scheme fails to provide adequate advice to product developers, users, and consumers. It will likely be very difficult, if not impossible, to incorporate NAMs into a Level 1 binary scheme. The result of using a Level 1 binary scheme would be to mischaracterize many substances. The binary approach would either over-interpret or under-interpret the NAM-based information resulting in an excess of substances requiring the unnecessarily stringent risk management that would be required under Level 1 classification or allow unrestricted use of potentially hazardous compounds . The well-accepted and current models of carcinogenicity support the development of schemes that will provide the basis of a scientifically robust process for assessing (potential) carcinogenicity. The application of new tools based on our current knowledge can be used to provide useful guidance to product developers, users, and consumers on the use of chemicals and prevent risk of excess cancer outcomes. The hazard banding approach to classification that incorporates MOA and potency presented here results in three easily understandable categories of potential carcinogenicity. Use of this scheme would result in placing chemicals into bands that would reflect an appropriate level of concern, which is not the case for Level 1 schemes. Each category leads to different generic advice on the use of the substances, including the currently accepted and stringent risk management measures as appropriate.

## Supplementary Information

Below is the link to the electronic supplementary material.

Supplementary file1 (DOCX 37 KB)
