# Supplementary material for: A new approach to the classification of carcinogenicity
Source: Arch Toxicol. 2022 Jun 15;96(9):2419–28. doi: 10.1007/s00204-022-03324-z (PMC9325845; doi:10.1007/s00204-022-03324-z)
Supplement: Supplementary file 1 — Supplementary file1 (DOCX 37 KB) [file 204_2022_3324_MOESM1_ESM.docx]

SUPPLEMENTARY DATA FOR

A New Approach to the Classification of Carcinogenicity

John E Doe^a^, Alan R Boobis^b^, Samuel M Cohen^c^, Vicki L Dellarco^d^, Penelope A Fenner-Crisp^e^, Angelo Moretto^f^, Timothy P Pastoor^g^, Rita S Schoeny^h^, Jennifer G Seed^i^, and Douglas C Wolf^j^

a School of Pharmacy and Biomolecular Sciences, Liverpool John Moores University, Byrom Street, Liverpool, L3 3AF, UK

b National Heart & Lung Institute, Hammersmith Campus, Imperial College London, London, W12 0NN, UK.

c Department of Pathology and Microbiology, Havlik-Wall Professor of Oncology, University of Nebraska Medical Center, Omaha, NE 68198-3135, USA.

d Independent Consultant, Silver Spring, MD 20901, USA.

e Independent Consultant, North Garden, VA 22959, USA

f Dipartimento di Scienze Cardio-Toraco-Vascolari e Sanità Pubblica (Department of Cardio-Thoraco-Vascular and Public Health Sciences), Università degli Studi di Padova, Padua, Italy

g Pastoor Science Communication, LLC, Greensboro, NC 27455, USA

h Rita Schoeny LLC, Washington DC, 20002, USA

i Independent Consultant, Alexandria, VA 22301, USA.

j Syngenta Crop Protection LLC, Greensboro, NC 27419, USA.

Corresponding Author: Dr John E Doe email: j.e.doe@ljmu.ac.uk

Carcinogenicity Evaluation

| Chemical |
| --- |
| Aflatoxin |
| Sources of Evidence |
| NIEHS Report on Carcinogens [RoC Profile: Aflatoxins; 15th RoC 2021 (nih.gov)](https://ntp.niehs.nih.gov/ntp/roc/content/profiles/aflatoxins.pdf)  Carcinogenicity of dietary aflatoxin M1 in male Fischer rats compared to aflatoxin B1  [J M Cullen](https://pubmed.ncbi.nlm.nih.gov/?term=Cullen+JM&cauthor_id=3102052), [B H Ruebner](https://pubmed.ncbi.nlm.nih.gov/?term=Ruebner+BH&cauthor_id=3102052), [L S Hsieh](https://pubmed.ncbi.nlm.nih.gov/?term=Hsieh+LS&cauthor_id=3102052), [D M Hyde](https://pubmed.ncbi.nlm.nih.gov/?term=Hyde+DM&cauthor_id=3102052), [D P Hsieh](https://pubmed.ncbi.nlm.nih.gov/?term=Hsieh+DP&cauthor_id=3102052) Cancer Res 1987 Apr 1;47(7):1913-7. |
| Lines of Evidence |
| Early evidence for the carcinogenicity of aflatoxins in humans came from epidemiological studies (a case-control study and descriptive studies) that correlated geographic variation in aflatoxin content of foods with geographic variation in the incidence of liver cancer (hepatocellular carcinoma, or primary liver-cell cancer). Studies in Uganda, Swaziland, Thailand, Kenya, Mozambique, and China demonstrated strong, significant positive correlations between estimated aflatoxin intake or aflatoxin levels in food samples and the incidence of liver cancer. In the United States, a 10% excess of primary liver-cell cancer was observed in the Southeast, where the estimated average daily intake of aflatoxin was high, compared with the North and West, areas with low aflatoxin intake. In a case-control study in the Philippines, levels of aflatoxin in the diets of individuals were estimated retrospectively, and the risk of liver cancer increased significantly with increasing estimated aflatoxin consumption. Interpretation of these studies is complicated by potential confounding due to hepatitis B virus infection, which is endemic in many of the study areas and is known to cause primary liver-cell cancer (IARC 1987, 1993)  Aflatoxins caused tumors in several species of experimental animals, at several different tissue sites, and by several different routes of exposure. Oral administration of aflatoxin mixtures or aflatoxin B1 alone (in the diet, by stomach tube, or in the drinking water) caused liver tumors (hepatocellular or cholangiocellular tumors) in all species tested except mice; these included rats, hamsters, marmosets, tree shrews, and monkeys. In addition, kidney (renal-cell) and colon tumors occurred in rats, benign lung tumors (adenoma) in mice, and tumors of the liver, bone (osteogenic sarcoma), gallbladder, and pancreas (adenocarcinoma) in monkeys. When administered by intraperitoneal injection, aflatoxin B1 caused liver tumors in infant mice, adult rats, and toads. Aflatoxin B1 administered by intraperitoneal injection to pregnant and lactating rats caused tumors of the liver, digestive tract, urogenital system, and nervous system in the mothers and offspring. Aflatoxin mixtures administered by subcutaneous injection caused tumors at the injection site (sarcoma) in rats and mice. Aflatoxins B2 , G1 , and M1 also caused liver tumors in experimental animals, but generally at lower incidences than did aflatoxin mixtures or aflatoxin B1 alone. In rats, aflatoxin G1 also caused kidney tumors when administered orally and a low incidence of injectionsite tumors (sarcoma) when administered by intraperitoneal injection. Both enhancement and inhibition of aflatoxin’s carcinogenicity were observed following co-administration of aflatoxins with various diets, viruses, parasites, known carcinogens, and other chemicals (IARC 1976, 1993). |
| Mode of Action |
| Aflatoxin causes genetic damage in bacteria, in cultured cells from humans and experimental animals, and in humans and experimental animals exposed to aflatoxin in vivo. Types of genetic damage observed include formation of DNA and albumin adducts, gene mutations, micronucleus formation, sister chromatid exchange, and mitotic recombination. Metabolically activated aflatoxin B1 specifically induced G to T transversion mutations in bacteria. G to T transversions in codon 249 of the p53 tumor-suppressor gene have been found in human liver tumors from geographic areas with high risk of aflatoxin exposure and in experimental animals (IARC 1993, 2002). In humans and susceptible animal species, aflatoxin B1 is metabolized by cytochrome P450 enzymes to aflatoxin-8,9-epoxide, a reactive form that binds to DNA and to albumin in the blood serum, forming adducts. Comparable levels of the major aflatoxin B1 adducts (the N7 -guanine and serum albumin adducts) have been detected in humans and susceptible animal species. The 8,9-epoxide metabolite can be detoxified through conjugation with glutathione, mediated by the enzyme glutathione S-transferase (GST). The activity of GST is much higher (by a factor of 3 to 5) in animal species that are resistant to aflatoxin carcinogenicity, such as mice, than in susceptible animal species, such as rats. Humans have lower GST activity than either mice or rats, suggesting that humans are less capable of detoxifying aflatoxin-8,9-epoxide. In studies of rats and trout, treatment with chemopreventive agents reduced the formation of aflatoxin B1 – guanine adducts and the incidence of liver tumors.  Primary (Direct) |
| Point of Departure |
| Aflatoxin M_1_ (AFM), an hydroxy metabolite of the potent carcinogenic mycotoxin aflatoxin B_1_ (AFB) is frequently found in milk and other dairy products. Sufficient amounts of AFM were produced to study the carcinogenicity of this compound. AFM was fed to male Fischer rats starting at 7 weeks up to 21 months of age. Agar-based semisynthetic diets contained 0.0, 0.5, 5.0, and 50.0 µg/kg of AFM or 50 µg/kg of AFB. Hepatocellular carcinomas were detected in two of 37 rats and neoplastic nodules were found in six of 37 rats fed 50 µg/kg AFM between 19 and 21 months. No nodules or carcinomas were observed in the lower AFM dose groups. Nineteen of 20 rats fed a diet containing 50 µg/kg of AFB developed hepatocellular carcinomas by 19 months of age. Carcinogenic potency of the aflatoxins was reflected by morphometric quantitation of foci detected in hematoxylin and eosin stained sections. Three rats fed the diet containing 50 µg/kg AFM developed intestinal carcinomas. None were observed in other groups. Under the conditions of this experiment AFM was found to be a weak hepatic carcinogen compared to AFB and to possess intestinal carcinogenicity  High Potency |
| Overall Category |
| Category A. Category A substances would require stringent risk management which may include the current EU “cut-off” approach used for plant protection products or be based on worst case assumptions of dose response (linear extrapolation, >10000 MoE).  IARC - |

Carcinogenicity Evaluation

| Chemical |
| --- |
| Benz(*a*)anthracene |
| Sources of Evidence |
| [Benz[a]anthracene (IARC Summary & Evaluation, Volume 32, 1983) (inchem.org)](https://inchem.org/documents/iarc/vol32/benz%5Ba%5Danthracene.html)  Certain Polycyclic Aromatic Hydrocarbons and Heterocyclic Compounds  IARC Monographs on the Evaluation of Carcinogenic Risk of Chemicals to Man Volume 3 |
| Lines of Evidence |
| Benz(a)anthracene (BA) and its five possible trans-dihydrodiols were evaluated for determination of their skin tumor-initiating activity and their mutagenic activity in Chinese hamster V79 cells. In addition, the skin tumor-initiating abilities of five diol-epoxides of BA were tested. Results showed (+/-)-trans-3,4-dihydroxy-3,4-dihydrobenz(a)anthracene (BA 3,4-dihydrodiol) to be approximately 10 times more mutagenic than was BA and about 20 times more mutagenic than were the other possible dihydrodiols in the V79 cells cocultivated with irradiated hamster embryo cells. As a skin tumor initiator, BA 3,4-dihydrodiol was approximately 5 times more active than BA, whereas the other BA dihydrodiols were all less active tumor initiators. (+/-)-trans-3alpha,4beta-Dihydroxy-1alpha,2alpha-epoxy-1,2,3,4-tetrahydrobenz(a)anthracene was found to be approximately 20% more active as a tumor initiator than was BA 3,4-dihydrodiol, whereas the other diol-epoxides of BA were less active than BA itself. The results suggest that the bay-region diol-epoxide of BA may be the ultimate carcinogen and mutagenic form of BA. |
| Mode of Action |
| Benz[*a*]anthracene was mutagenic to *Salmonella typhimurium* in the presence of an exogenous metabolic system and was mutagenic to *Drosophila melanogaster*; it was also mutagenic to mammalian cells *in vitro* in the presence of an exogenous metabolic system. This compound was positive in one study of sister chromatid exchange. It induced unscheduled DNA synthesis in cultured mammalian cells and morphological transformation. In one in-vivo study, it induced sister chromatid exchange in hamsters; reports from in-vivo studies on the induction of chromosomal aberrations were conflicting.  Primary (Direct) |
| Point of Departure |
| Single application of 2µmol  High |
| Overall Category |
| Category A - would require stringent risk management which may include the current EU “cut-off” approach used for plant protection products or be based on worst case assumptions of dose response (linear extrapolation, >10000 MoE).  IARC 2B |

Carcinogenicity Evaluation

| Chemical |
| --- |
| B-Raf inhibitors |
| Sources of Evidence |
| Toxicol Pathol 2011 Aug;39(5):809-22.  **Raf inhibition causes extensive multiple tissue hyperplasia and urinary bladder neoplasia in the rat** [John A Wisler](https://pubmed.ncbi.nlm.nih.gov/?term=Wisler+JA&cauthor_id=21677315) [^1^](https://pubmed.ncbi.nlm.nih.gov/21677315/#affiliation-1), [Cynthia Afshari](https://pubmed.ncbi.nlm.nih.gov/?term=Afshari+C&cauthor_id=21677315), [Mark Fielden](https://pubmed.ncbi.nlm.nih.gov/?term=Fielden+M&cauthor_id=21677315), [Cameron Zimmermann](https://pubmed.ncbi.nlm.nih.gov/?term=Zimmermann+C&cauthor_id=21677315), [Scott Taylor](https://pubmed.ncbi.nlm.nih.gov/?term=Taylor+S&cauthor_id=21677315), [Josette Carnahan](https://pubmed.ncbi.nlm.nih.gov/?term=Carnahan+J&cauthor_id=21677315), [Steven Vonderfecht](https://pubmed.ncbi.nlm.nih.gov/?term=Vonderfecht+S&cauthor_id=21677315)  PMID: 21677315 DOI: [10.1177/0192623311410442](https://doi.org/10.1177/0192623311410442) |
| Lines of Evidence |
| Seven novel and potent Raf small molecule kinase inhibitors (C1-7) were evaluated in seven-day oral repeat dose rat toxicity studies. All compounds tested induced hyperplasia in multiple tissues. Consistently affected was stratified squamous epithelium at a number of sites and transitional epithelium of urinary bladder and kidney. A seven-day time course study in rats showed morphologic evidence of epithelial proliferation in the nonglandular stomach within four to five hours after a single dose of C-1. Similar indications of cellular proliferation were observed in the urinary bladder by day 2 and in the heart, kidney, and liver by day 3. Transcriptional evidence of proliferation in the urinary bladder was detected within four to five hours after a single dose consistent with activation of the PI3K/AKT and ERK/MAPK pathways. In a twenty-eight-day rat toxicity study of C-1, hyperplasia was observed in the esophagus, nonglandular stomach, skin, urinary bladder, kidney, and heart. Hyperplasia of transitional epithelium of the urinary bladder was particularly severe and in one female rat was accompanied by the presence of a transitional cell carcinoma. |
| Mode of Action |
| These results suggest that these Raf inhibitors induce early transcriptional changes driving unchecked cell proliferation, resulting in marked tissue hyperplasia that can progress to carcinoma within a short time frame.  Secondary (Indirect) |
| Point of Departure |
| Effect level 30mg/kg for 28 days  High Potency |
| Overall Category |
| A |

Carcinogenicity Evaluation

| Chemical |
| --- |
| Dicloroethane |
| Sources of Evidence |
| [Registration Dossier - ECHA (europa.eu)](https://echa.europa.eu/registration-dossier/-/registered-dossier/15430/7/7/1) |
| Lines of Evidence |
| Oral:  Two long-term carcinogenicity studies in rat and mice showed tumours in a number of organs (mammary gland adenocarcinomas, squamous cell carcinomas of the forestomach and haemangiosarcomas) after oral 1,2-dichloroethane administration by gavage.  Inhalation:  A 2-yr inhalation exposure of rats and mice to 1,2-dichloroethane produced a dose-dependent increase in incidences of benign and malignant tumours.  Carcinogenicity and chronic toxicity of 1,2- dichloroethane were examined by inhalation exposure of groups of 50 F344 rats of both sexes to 1,2- dichloroethane vapor or clean air as control for 6 h/d, 5 d/wk and 104 wk. The rats were exposed to 0, 10, 40 or 160 ppm (v/v) (equivalent to 0, 41.1, 164.5 or 658.1 mg/m^3^) 1,2- dichloroethane. The 2-yr exposure to 1,2- dichloroethane produced a dose-dependent increase in incidences of benign and malignant tumors, including subcutaneous fibroma, mammary gland fibroadenoma and peritoneal mesothelioma in male rats; subcutaneous fibroma and mammary gland adenoma, fibroadenoma and adenocarcinoma in female rats. No exposure-related change in the incidence of non-neoplastic lesions or in any hematological, blood biochemical or urinary parameter occurred in any 1,2- dichloroethane -exposed rat group. Selection of the exposure concentrations was considered appropriate with reference to the maximum tolerated dose for the highest doses and an occupational exposure limit of 1,2 - dichloroethane for the lowest dose. A BMC10 and T25 value of 42 ppm and 99 ppm, repsectively, have been derived (obtained for the modelling of combined fibroadenoma and adenoma and combined fibroadenoma, adenoma and adenocarcinoma in female rats, respectively).  There is evidence that 1,2-dichloroethane interacts with DNA in exposed animals in the absence of overt toxicity. The *in vivo*effects include DNA strand breaks and observations of DNA binding at the macromolecular level and are dose-dependent and systemic, with multiple organs involved. However, many of these assays are limited for assessing the genotoxic potential of 1,2-dichloroethane due to the use of high doses and/or an inadequate study design. Limited DNA damage and/or binding was demonstrated following inhalation exposure compared to oral and intraperitoneal exposures.  The results of the available *in vivo*studies failed to show a mutagenic potential of 1,2-dichloroethane as the substance was clearly negative in 3 micronucleus assays (2 by i.p. and one repeated dosing by the oral route) and in a dominant lethal test with repeated exposure on 2 generations. Some evidence of DNA interaction is presented by positive results in an SCE assay (a non-mutagenic endpoint), in DNA strand-break assays, and in a test with Drosophila. Moreover, the ability of 1,2-dichloroethane to produce genotoxic effects in germ cells of male mice was considered unlikely. In particular, a recently conducted Comet assay in mammary gland epithelial cells (the target organ for carcinogenicity) after *in vivo*inhalation exposure of female rats to 200 ppm 1,2-dichloroethane for 4 weeks was clearly negative. |
| Mode of Action |
| No exposure-related change in the incidence of non-neoplastic lesions or in any hematological, blood biochemical or urinary parameter occurred in any 1,2- dichloroethane -exposed rat group.  Based on a weight-of-evidence approach it is concluded that 1,2-dichloroethane is not mutagenic *in vivo*. The results of the available mutagenicity and genotoxicity assays do not provide convincing support for a genotoxic mode of action for the carcinogenicity of 1,2-dichloroethane.  Non-genotoxic but no evidence for Tertiary (Collateral). Assume Secondary (Indirect) |
| Point of Departure |
| LOAEL 47mg/kg/day Medium Potency |
| Overall Category |
| Category B Category B substances would require health based guidance values on the effect which leads to the modification of carcinogenesis for appropriate risk management.  EU Category 1B  IARC 2B |

Carcinogenicity Evaluation

| Chemical |
| --- |
| Hydroquinone |
| Sources of Evidence |
| McGregor Crit Rev Toxicol 2007;37(10):887-914. doi: 10.1080/10408440701638970. |
| Lines of Evidence |
| The toxicology of hydroquinone has been reviewed on a number of previous occasions. This review targets its potential for carcinogenicity and possible modes of carcinogenic action. The evaluation made by IARC (1999) of its carcinogenic risk to humans was that hydroquinone is not classifiable as to its carcinogenicity to humans (Group 3). This evaluation was based on inadequate evidence in humans and limited evidence in experimental animals. The epidemiological information comes from four cohort studies involving occupational exposures. A cohort of lithographers, some of whom had worked with hydroquinone, had an excess of malignant melanoma based on five cases, but only two of the cases had reported exposure to hydroquinone. In a study of photographic processors the number of exposed individuals was uncertain and the numbers of cases of individual cancer sites were small. In view of the statistical power limitations of these studies for individual diagnostic categories of cancers, they are not considered to be informative with regard to the carcinogenicity of hydroquinone. A cohort of workers with definite and lengthy exposure to hydroquinone, during either its manufacture or its use, had low cancer rates compared with two comparison populations; the reason for the lower than expected rates is unclear. In a motion picture film processing cohort there were significant excess malignancies of the respiratory system among workers engaged in developing, where there was exposure to hydroquinone as well as other chemicals. There was no information on tobacco smoking habits and no dose-response relationship. Hydroquinone has been shown reproducibly to induce benign neoplasms in the kidneys of male F344 rats dosed orally either by gavage (25 and 50 mg/kg body weight) or diet (0.8%). The gavage study has been evaluated in considerable detail. This evaluation showed that all renal tubule adenomas and all cases of renal tubule atypical hyperplasia occurred in areas of severe or end-stage chronic progressive nephropathy and that the neoplasms were not otherwise confined to any particular part of the kidney. It is likely that the mode of carcinogenic action of hydroquinone is exacerbation of this natural disease process. Hydroquinone is mutagenic in vitro and in vivo, having caused genotoxicity or chromosomal aberrations in rodent bone-marrow cells. At least a portion, if not all, of the chromosomal effects are caused by interference by hydroquinone or its metabolites with chromosomal segregation, probably due to interaction with mitotic spindle proteins. However, the dose routes used to demonstrate these effects in almost all of the studies in vivo were intraperitoneal or subcutaneous injection, which were considered inappropriate. There were five studies by the oral route. These included a mouse bone-marrow cell micronucleus test in which a weak, marginally positive response was obtained following a single oral dose of 80 mg/kg body weight. The remaining oral route studies all showed no significant effect. They included a mouse bone-marrow cell micronucleus test in which there was no genotoxic activity after exposure to a diet containing 0.8% hydroquinone for 6 days; two (32)P-post-labeling assays, one with targets of Zymbal gland, liver, and spleen in Sprague-Dawley rats, the other with the kidney as target in F344 rats; and the last oral assay was for 8-hydroxydeoxyguanosine adducts in F344 rat kidney DNA. Thus, the evidence (and the database) for any genotoxic effect in vivo is sparse and none has been observed in kidney. While glutathione conjugates could be responsible for the tumor induction, careful histology seems to show that the most actively toxic of several glutathione compounds tested, 2,3,5-triglutathion-S-yl hydroquinone, targets a very specific region of the kidney, the outer stripe of the outer medulla (OSOM), whereas hydroquinone-associated adenomas are more randomly distributed and occur in the cortex as well as the medulla. A nongenotoxic mode of action that involves exacerbation of a spontaneously occurring rodent renal disease, chronic progressive nephropathy (CPN), is proposed and evaluated. This disease is particularly prominent in male rats and the evidence is consistent with an absence of any human counterpart; therefore, the increased incidence of renal tubule adenomas in hydroquinone-dosed male rats is without human consequence. |
| Mode of Action |
| A nongenotoxic mode of action that involves exacerbation of a spontaneously occurring rodent renal disease, chronic progressive nephropathy (CPN) Tertiary (Collateral) |
| Point of Departure |
| LOAEL 25mg/kg Medium Potency |
| Overall Category |
| Category C  Category C substances require risk management determined from health-based guidance values based on repeat dose toxicity  Current EU classification – Category 2  IARC 3  US EPA: Group C without Q1* |

Carcinogenicity Evaluation

| Chemical |
| --- |
| Linuron |
| Sources of Evidence |
| [Peer review of the pesticide risk assessment of the active substance linuron (wiley.com)](https://efsa.onlinelibrary.wiley.com/doi/epdf/10.2903/j.efsa.2016.4518) |
| Lines of Evidence |
| As from DAR summary evaluation: Linuron undergoes metabolic activation, is not genotoxic, induces oxidative stress and protein reaction (methaemoglobin), inflammatory response, cell proliferation, cell death (cytotoxicity)  In **long-term toxicity and carcinogenicity** studies with rats, the critical effects at the agreed lowest observable adverse effect level (LOAEL) of 1.3 mg/kg bw per day included decreased incidence of pituitary adenoma/carcinoma and equivocal increase of Leydig cell adenoma in male rats. No NOAEL could be identified in rats. In mice, the long-term NOAEL is 6.5 mg/kg bw per day based on reduced body weight gain in males. Increased incidence of Leydig cell tumours, uterine adenocarcinoma and ovarian (granulosa/thecal cell) tumours was also observed at higher dose levels in rats whereas an increase in hepatocellular adenoma was observed in mice.  Mechanistic studies indicated that linuron is an anti-androgenic compound,  Non-genotoxic |
| Mode of Action |
| Antiandrogen. Tumour induction probably caused by increased cell division as a compensatory mechanism for increased androgen production. Tertiary (collateral) |
| Point of Departure |
| 6.5mg/kg/day NOAEL in mice Medium potency |
| Overall Category |
| Category C  Category C substances require risk management determined from health-based guidance values based on repeat dose toxicity  Linuron is currently classified as carcinogenic category 2 and as toxic for reproduction category 1B, |

Carcinogenicity Evaluation

| Chemical |
| --- |
| Ochratoxin A |
| Sources of Evidence |
| Toxicol Appl Pharmacol  . 2014 Nov 1;280(3):543-9.   doi: 10.1016/j.taap.2014.08.030. Epub 2014 Sep 8.  **Ochratoxin A induces rat renal carcinogenicity with limited induction of oxidative stress responses**  [Xiaozhe Qi](https://pubmed.ncbi.nlm.nih.gov/?term=Qi+X&cauthor_id=25218026) [^1^](https://pubmed.ncbi.nlm.nih.gov/25218026/#affiliation-1), [Tao Yu](https://pubmed.ncbi.nlm.nih.gov/?term=Yu+T&cauthor_id=25218026) [^1^](https://pubmed.ncbi.nlm.nih.gov/25218026/#affiliation-1), [Liye Zhu](https://pubmed.ncbi.nlm.nih.gov/?term=Zhu+L&cauthor_id=25218026) [^1^](https://pubmed.ncbi.nlm.nih.gov/25218026/#affiliation-1), [Jing Gao](https://pubmed.ncbi.nlm.nih.gov/?term=Gao+J&cauthor_id=25218026) [^1^](https://pubmed.ncbi.nlm.nih.gov/25218026/#affiliation-1), [Xiaoyun He](https://pubmed.ncbi.nlm.nih.gov/?term=He+X&cauthor_id=25218026) [^2^](https://pubmed.ncbi.nlm.nih.gov/25218026/#affiliation-2), [Kunlun Huang](https://pubmed.ncbi.nlm.nih.gov/?term=Huang+K&cauthor_id=25218026) [^2^](https://pubmed.ncbi.nlm.nih.gov/25218026/#affiliation-2), [Yunbo Luo](https://pubmed.ncbi.nlm.nih.gov/?term=Luo+Y&cauthor_id=25218026) [^2^](https://pubmed.ncbi.nlm.nih.gov/25218026/#affiliation-2), [Wentao Xu](https://pubmed.ncbi.nlm.nih.gov/?term=Xu+W&cauthor_id=25218026) [^3^](https://pubmed.ncbi.nlm.nih.gov/25218026/#affiliation-3)   - PMID: 25218026 - DOI: [10.1016/j.taap.2014.08.030](https://doi.org/10.1016/j.taap.2014.08.030)   Chem Res Toxicol  . 2012 Feb 20;25(2):252-62.   doi: 10.1021/tx200430f. Epub 2011 Nov 16.  **An update on direct genotoxicity as a molecular mechanism of ochratoxin a carcinogenicity**  [Annie Pfohl-Leszkowicz](https://pubmed.ncbi.nlm.nih.gov/?term=Pfohl-Leszkowicz+A&cauthor_id=22054007) [^1^](https://pubmed.ncbi.nlm.nih.gov/22054007/#affiliation-1), [Richard A Manderville](https://pubmed.ncbi.nlm.nih.gov/?term=Manderville+RA&cauthor_id=22054007)  Affiliations expand   - PMID: 22054007 - DOI: [10.1021/tx200430f](https://doi.org/10.1021/tx200430f)     Mol Nutr Food Res  . 2007 Jan;51(1):61-99.   doi: 10.1002/mnfr.200600137.  **Ochratoxin A: An overview on toxicity and carcinogenicity in animals and humans**  [Annie Pfohl-Leszkowicz](https://pubmed.ncbi.nlm.nih.gov/?term=Pfohl-Leszkowicz+A&cauthor_id=17195275) [^1^](https://pubmed.ncbi.nlm.nih.gov/17195275/#affiliation-1), [Richard A Manderville](https://pubmed.ncbi.nlm.nih.gov/?term=Manderville+RA&cauthor_id=17195275)   - PMID: 17195275 - DOI: [10.1002/mnfr.200600137](https://doi.org/10.1002/mnfr.200600137)   **Erratum in**   - Mol Nutr Food Res. 2007 Sep;51(9):1192 |
| Lines of Evidence |
| Ochratoxin A (OTA) has displayed nephrotoxicity and renal carcinogenicity in mammals, however, no clear mechanisms have been identified detailing the relationship between oxidative stress and these toxicities. This study was performed to clarify the relationship between oxidative stress and the renal carcinogenicity induced by OTA. Rats were treated with 70 or 210 μg/kg b.w. OTA for 4 or 13 weeks. In the rats administrated with OTA for 13 weeks, the kidney was damaged seriously.  Ochratoxin A (OTA) is a ubiquitous mycotoxin produced by fungi of improperly stored food products. OTA is nephrotoxic and is suspected of being the main etiological agent responsible for human Balkan endemic nephropathy (BEN) and associated urinary tract tumours. Striking similarities between OTA-induced porcine nephropathy in pigs and BEN in humans are observed. International Agency for Research on Cancer (IARC) has classified OTA as a possible human carcinogen (group 2B). Currently, the mode of carcinogenic action by OTA is unknown. OTA is genotoxic following oxidative metabolism. This activity is thought to play a central role in OTA-mediated carcinogenesis and may be divided into direct (covalent DNA adduction) and indirect (oxidative DNA damage) mechanisms of action. Evidence for a direct mode of genotoxicity has been derived from the sensitive 32P-postlabelling assay. OTA facilitates guanine-specific DNA adducts in vitro and in rat and pig kidney orally dosed, one adduct comigrates with a synthetic carbon (C)-bonded C8-dG OTA adduct standard. In this paper, our current understanding of OTA toxicity and carcinogenicity are reviewed. The available evidence suggests that OTA is a genotoxic carcinogen by induction of oxidative DNA lesions coupled with direct DNA adducts via quinone formation. This mechanism of action should be used to establish acceptable intake levels of OTA from human food sources. |
| Mode of Action |
| In the rats administrated with OTA for 13 weeks, the kidney was damaged seriously. Cytoplasmic vacuolization was observed in the outer stripe of the outer medulla. Karyomegaly was prominent in the tubular epithelium. Kidney injury molecule-1 (Kim-1) was detected in the outer stripe of the outer medulla in both low- and high-dose groups. OTA increased the mRNA levels of clusterin in rat kidneys. Interestingly, OTA did not significantly alter the oxidative stress level in rat liver and kidney. Yet, some indications related to proliferation and carcinogenicity were observed. A dose-related increase in proliferating cell nuclear antigen (PCNA) was observed at 4 weeks in both liver and kidney, but at 13 weeks, only in the kidney. OTA down-regulated reactive oxygen species (ROS) and up-regulated vimentin and lipocalin 2 in rat kidney at 13 weeks. The p53 gene was decreased in both liver and kidney at 13 weeks. These results suggest that OTA caused apparent kidney damage within 13 weeks but exerted limited effect on oxidative stress parameters. It implies that cell proliferation is the proposed mode of action for OTA-induced renal carcinogenicity.  Ochratoxin A (OTA) is a naturally occurring chlorophenolic fungal toxin that contaminates a wide range of food products and poses a cancer threat to humans. The mechanism of action (MOA) for OTA renal carcinogenicity is a controversial issue. In 2005, direct genotoxicity (covalent DNA adduct formation) was proposed as a MOA for OTA-mediated carcinogenicity [ Manderville , R. A. ( 2005 ) Chem. Res. Toxicol. 18 , 1091 - 1097 ]. At that time, inconsistent results had been published on OTA genotoxicity/mutagenicity, and conclusive evidence for OTA-mediated DNA adduction had been lacking. In this update, published data from the past 6-7 years are presented that provide new hypotheses for the MOA of OTA-mediated carcinogenicity. While direct genotoxicity remains a controversial issue for OTA, new findings from the Umemura and Nohmi laboratories provide definitive results for the mutagenicity of OTA in the target tissue (outer medulla) of male rat kidney that rules out oxidative DNA damage. These findings, coupled with our own efforts that provide new structural evidence for DNA adduction by OTA, has strengthened the argument for involvement of direct genotoxicity in OTA-mediated renal carcinogenesis. This MOA should be taken into consideration for OTA human risk assessment.  Collateral (Tertiary) |
| Point of Departure |
| Activity at 70ug/kg  High Potency |
| Overall Category |
| B - would require health based guidance values on the effect which leads to the modification of carcinogenesis for appropriate risk management.  IARC 2B |

Carcinogenicity Evaluation

| Chemical |
| --- |
| Titanium Dioxide |
| Sources of Evidence |
| [Identification of research needs to rsolve the carcinogenicity of high-priority IARC carcinogens (who.int)](https://monographs.iarc.who.int/wp-content/uploads/2018/06/TR42-4.pdf). Eileen D. Kuempel PhD and Avima Ruder PhD |
| Lines of Evidence |
| Lee et al. (1985) Rats (female CD Sprague-Dawley-derived) were exposed by whole body inhalation to fine, rutile TiO2 (aerodynamic mass median diameter of 1.5-1.7 µm) for 6 hr/day, 5 days/week, for 32 up to two years, to 0, 10, 50, or 250 mg/m3 (84% respirable; <13 µm MMAD); 80 rats were exposed for two years, and all surviving rats were killed at the end of exposure. No increase in lung tumors was observed at 10 or 50 mg/m3 . At 250 mg/m3 , bronchioalveolar adenomas were observed in 12/77 male rats and 13/74 female rats. In addition, squamous cell carcinomas were reported in 1 male and 13 females at 250 mg/m3 . These squamous cell carcinomas were later reclassified as proliferative keratin cysts (Carlton 1994), or as a range of responses from pulmonary keratinizing cysts through pulmonary keratinizing eptheliomas to frank pulmonary squamous carcinomas (Boorman et al., 1996). A recent reanalysis of the 16 tumors originally classified as cystic keratinizing squamous cell carcinomas in Lee et al. (1985) had a similar interpretation: two were re-classified as squamous metaplasia, one as a poorly keratinizing squamous cell carcinoma, and 13 as nonneoplastic pulmonary keratin cysts (Warheit and Frame 2006).  Heinrich et al. (1995) Female Wistar rats were exposed to ultrafine TiO2 (80% anatase/20% rutile; 15-40 nm primary particle size; 0.8 μm MMAD; 48 (+ 2.0) m2 /g specific surface area) at an average concentration of 10 mg/m3 , 18 h/d, 5d/wk, for up to 24 months (actual concentrations were 7.2 mg/m3 for 4 months, followed by 14.8 mg/m3 for 4 months, and 9.4 mg/m3 for 16 months). After the 2-year exposure, the rats were kept in clean air for an additional 6 months. After 24 months of exposure, four of the nine rats examined had developed tumors (including a total of 2 squamous cell carcinomas, 1 adenocarcinoma, and 2 benign squamous cell tumors). At 30 months (6 months after the end of exposure), a statistically significant increase in adenocarcinomas was observed (13 adenocarcinomas, in addition to 3 squamous cell carcinomas and 4 adenomas, in 100 rats). In addition, 20 rats had benign keratinizing cystic squamous-cell tumors. Only 1 adenocarcinoma, and no other lung tumors, was observed in 217 nonexposed control rats. |
| Mode of Action Conclusion |
| Tertiary (Collateral)  Titanium dioxide is poorly soluble low toxicity (PSLT) particles, which can elicit overloading of lung clearance, chronic inflammation, and lung tumors in rats following prolonged exposure at sufficiently high concentrations of particles (Monograph 93 (in press); Baan 2007). Overloading of lung clearance occurs at much lower mass concentrations of ultrafine TiO2 (10 mg/m3 ) than fine TiO2 (50 or 250 mg/m3 ) (Bermudez et al., 2002, 2004). Lung tumors also develop a lower mass concentration of ultrafine TiO2 (~10 mg/m3 ) (Heinrich et al., 1995) compared to fine TiO2 (250 mg/m3 ) (Lee et al., 1985) following chronic inhalation in rats. Particle surface area dose was found to be most predictive of the pulmonary inflammation and tumor responses in rats when the dose-response relationships are compared for various types and sizes of PSLT including TiO2 (Driscoll 1995; Dankovic et al., 2007).  Most evidence suggests that TiO2 and other PSLT-elicited lung tumors develop via a secondary genotoxic mechanism involving chronic inflammation, cell proliferation, and oxidative stress (Schins and Knaapen 2007). Overloading of lung clearance is accompanied by pulmonary inflammation, production of reactive oxygen and nitrogen species, depletion of antioxidants and/or impairment of other defense mechanisms, cell injury, cell proliferation, fibrosis, and as observed in rats, induction of mutations and eventually cancer (Monograph 93 (in press); Baan 2007). |
| Potency |
| Medium Potency  NOAEL 50mg/m^3^ |
| Overall Category |
| Category C  Category C substances require risk management determined from health-based guidance values based on repeat dose toxicity  Current EU classification – Category 2  IARC 2B |

Carcinogenicity Evaluation

| Chemical |
| --- |
| Trichloroethylene |
| Sources of Evidence |
| [Fin Rep May 2014 (europa.eu)](https://echa.europa.eu/documents/10162/13641/tce_carcinogenity_dose-response_analysis_final_report_en.pdf/fbcd4b8d-eab7-4788-bef5-8815b584c5ff#:~:text=trichloroethylene%20exposure%20is%20very%20complex%2C%20involving%20multiple%20genotoxic,carcinogen%20due%20to%20data%20on%20the%20mutagenicity%20of) |
| Lines of Evidence |
| The purpose of this evaluation was to derive a WHO drinking-water quality guideline on trichloroethylene. The basis for evaluating the carcinogenicity in experimental animals was to a great extent the same as in the WHO (2000) evaluation, as no newer studies were included; however, emphasis was put on the oral studies (NTP 1983; NTP 1988 and NTP 1990). WHO (2005) put further emphasis on the findings of kidney tumours in rats to be relevant for humans compared to the WHO (2000) evaluation. Also the section regarding epidemiological data was much more extended than the WHO (2000) evaluation, and considerations regarding carcinogenic mode of action were described in more detail. The experimental animal results considered most pertinent by WHO (2005) in assessing the weight of evidence of carcinogenicity of trichloroethylene in humans were principally the significant increases in kidney tumours in rats (NTP, 1983, 1990), pulmonary tumours in mice (Fukuda et al., 1983; Maltoni et al., 1986, 1988; NTP, 1988) and testicular tumours in rats (Maltoni et al., 1986, 1988; NTP, 1988). Although there was some doubt about the human relevance of pulmonary tumours in mice, the potential for lung tumours in humans could not be ruled out. |
| Mode of Action |
| Various mode of actions were considered e.g.: non-genotoxic processes related to cytotoxicity, peroxisome proliferation and altered cell signalling; genotoxic processes, such as the production of genotoxic metabolites (e.g., chloral and DCVC); or the production of reactive oxygen species related to peroxisomal induction in the liver. As trichloroethylene appeared to be weakly genotoxic in in vitro and in vivo assays, and as several mutagenic or carcinogenic metabolites were formed, the genotoxic mode of action was not to be ignored. The evidence surrounding kidney tumours in rats after oral exposure was especially considered. Although it was noted that the tumours were few, the finding was repeatable in Sprague-Dawley rats exposed to trichloroethylene by the inhalational route. The tumours are historically rare in rats, so their appearance among dosed animals was considered biologically significant. Also, there were similarities between sites and histopathological characteristics of the tumours observed in human patients and in rat bioassays. The metabolites derived from trichloroethylene were noted to be identical in humans and in experimental animals. So, small increases in renal tumours in male rats at doses inducing renal damage were found to support the epidemiological evidence. Further support regarding mode of action was noted from the data on multiple mutations of the von Hippel Landau tumour suppressor genes followed by renal neoplasia that has been found in renal carcinoma patients with high prolonged trichloroethylene exposure.  Direct (Primary) |
| Point of Departure |
| 500mg/kg NOAEL in NTP assay  Low Potency |
| Overall Category |
| B - would require health based guidance values on the effect which leads to the modification of carcinogenesis for appropriate risk management.  IARC – 1  EU/GHS – 1B |
